# Supplementary material for: Platycodigenin as Potential Drug Candidate for Alzheimer’s Disease via Modulating Microglial Polarization and Neurite Regeneration
Source: Molecules. 2019 Sep 4;24(18):3207. doi: 10.3390/molecules24183207 (PMC6767002; doi:10.3390/molecules24183207)
Supplement: Supplementary file 1 [file molecules-24-03207-s001.pdf]

Platycodigenin as Potential Drug Candidate for Alzheimer's Disease via Modulating Microglial Polarization and Neurite Regeneration

Zhiyou Yang [1,2,*], Baiping Liu [1], Long-en Yang [1] and Cai Zhang [1]

[1] College of Food Science and Technology, Guangdong Provincial Key Laboratory of Aquatic Product Processing and Safety, Institute of nutrition and marine drugs, Guangdong Ocean University, Zhanjiang 524088, China; 15602575661@163.com(B.L.); yanglongen123@163.com (L.Y.); zhangcai910206@163.com (C.Z.)

[2] Shenzhen Institute of Guangdong Ocean University, Shenzhen 518120, China

* Correspondence: yang_zhiyou@sina.com; Tel.: +86-075-9239-6046

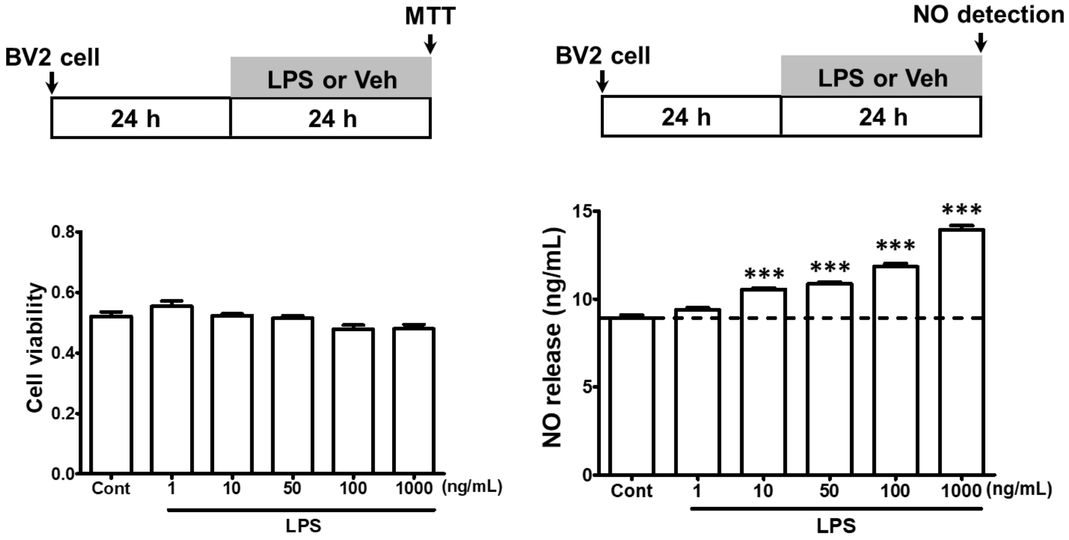

Figure S1. Effects of LPS on cell viability and NO production in BV2 microglia. BV2 microglia (2000 cells/well) were seeded in 96-well plates for 24 h, followed by treatment with LPS for 24 h and the cell viability was assayed. BV2 microglia (2×10^5 cells/mL) were seeded in 96-well plates for 24 h, followed by treatment with LPS for 24 h. NO release was detected by Griess reagent method.
